# Red blood cell distribution width is associated with neuronal damage in acute ischemic stroke

**DOI:** 10.18632/aging.103250

**Published:** 2020-05-23

**Authors:** Rong-Hua Hong, Jian Zhu, Ze-Zhi Li, Jian Yuan, Pei Zhao, Jie Ding, Qing-Lei Fan, Jin Yang, Bao-Guo Liu, Jian Cai, De-Sheng Zhu, Yang-tai Guan

**Affiliations:** 1 Department of Neurology, Baoshan Branch, Renji Hospital, School of Medicine, Shanghai Jiaotong University, Shanghai 200444, China; 2 Department of Neurology, Renji Hospital, School of Medicine, Shanghai Jiaotong University, Shanghai 200127, China

**Keywords:** acute ischemic stroke, red blood cell distribution width, neuron-specific enolase, multivariate analysis

## Abstract

Elevated red blood cell distribution width (RDW) has been found to be associated with the occurrence of ischemic stroke. However, there is no defined relationship between RDW and neuronal damage in acute ischemic stroke (AIS). This study was designed to determine the relationship between RDW and neuronal damage in AIS patients. A total of 442 consecutive AIS patients from January 2018 to June 2019 were evaluated for neuronal damage, which was estimated by serum neuron-specific enolase (NSE) levels. Red blood cell distribution width-standard deviation (RDW-SD), a parameter that reflects the heterogeneity of red blood cell volume, was also assessed. We evaluated the association between the RDW-SD and serum NSE level through multivariate-adjusted linear regression analysis. Both the serum NSE level and the incidence of high NSE increased according to the increased RDW-SD tertile in AIS patients (*p*<0.01). There was a positive correlation between RDW-SD and serum NSE levels (*r*=0.275, 95% CI: 0.187-0.359, *p*<0.001). The beta coefficients (95% CI) between RDW-SD and serum NSE levels were 0.32 (0.21-0.42, *p*<0.001) and 0.26 (0.15-0.38, *p*<0.001), respectively, in AIS patients before and after adjusting for potential confounders. In conclusion, we found a significant positive association between RDW-SD and neuronal damage in AIS patients.

## INTRODUCTION

The red blood cell volume distribution width (RDW) is a parameter reflecting the volume heterogeneity of peripheral blood red blood cells [1]. RDW was initially used to distinguish between types of anemia. In recent years, many studies have revealed that RDW is an independent risk factor for many critical clinical cases, especially for cardiovascular and cerebrovascular diseases [2, 3]. Furthermore, elevated RDW has been associated with thrombotic disorders, including myocardial infarction, venous thromboembolism, ischemic stroke, and hemorrhagic transformation, and these associations are independent of other inflammatory and coagulation biomarkers [4–7]. In clinical practice, red blood cell distribution width-standard deviation (RDW-SD) is often used to accurately reflect the volume variation of red blood cells.

Neuron-specific enolase (NSE), an enolase involved in glycolysis, was first described by Moore and McGregor in 1965 [8]. NSE exists in nerve tissue and neuroendocrine tissue, and the highest levels are found in central nerve cells. When the blood-brain barrier of the central nervous system is damaged, NSE is released into the blood circulation [9]. Several previous studies have suggested that serum NSE is associated with infarction volume, worse outcome, hemorrhagic transformation, and other neurological and neuroendocrine diseases [10–13]. Furthermore, serum NSE showed high predictive value for determining severity and early neurobehavioral outcome in acute ischemic stroke (AIS) patients [14–16]. Therefore, serum NSE has been used as a valuable neurobiochemical protein marker and is widely applied in the assessment of neuronal damage in patients with cerebrovascular diseases [17].

However, previous retrospective and prospective observational studies on RDW were limited to the occurrence of ischemic stroke. In addition, there is no defined relationship between RDW and neuronal damage in AIS patients. Based on current evidence, the association between RDW-SD and serum NSE levels remain unclear in the AIS population, and whether elevated RDW-SD levels are an important risk factor for serum NSE in the AIS population needs to be further investigated. Thus, we conducted this cross-sectional study, aiming to assess the association between RDW-SD and serum NSE levels in the AIS population. To our knowledge, no study has previously examined the association between the RDW-SD level and serum NSE in the AIS population.

## RESULTS

### Baseline characteristics

**Figure 1:**
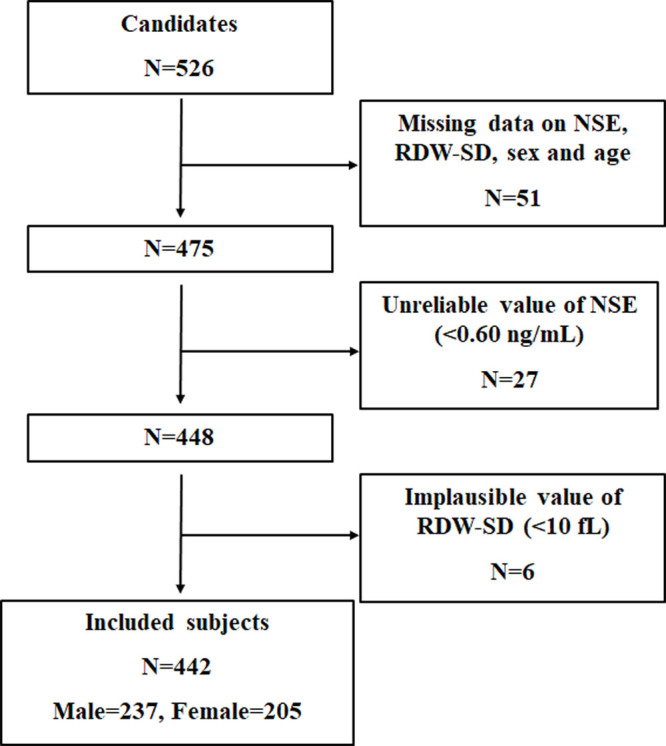
**Flowchart of the study.**

A total of 526 consecutive candidates were recruited for the study at the time of the final survey in July 2019. Among these candidates, those who had missing data related to serum NSE, RDW-SD, sex and age were excluded from the eligible candidates for this study (n=51). Those with unreliable values of serum NSE (<0.60 nm/mL) (n=27) and those with implausible values of RDW-SD (<10 fL) (n=6) were also excluded from the pool of eligible candidates for this study. As a result, a total of 442 subjects were included for the final analyses. A flowchart of the study is shown in Figure 1.

Among 442 study subjects, male accounted for 53.62% (n=237) and female accounted for 46.38% (n=205). The age of the enrolled subjects ranged from 40 to 99 yr (female, 49–99 yr; male, 40–91 yr), with a mean age of 73.11±10.80 yr (female, 76.50±10.19 yr; male, 70.18±10.47 yr). The disease duration before admission ranged from 0.5-46 hours, and the value was abnormally distributed with a median and interquartile range of 7 (4-17.75) hours. The RDW-SD ranged from 35.2 to 57.5 fL, and the NSE ranged from 6.56 to 23.82 ng/mL. The mean RDW-SD was significantly higher in the NSE high group than it was in the NSE normal group (43.49±3.80 fL *vs* 41.67±2.45 fL, *p*<0.001), and hierarchical analysis by gender showed significant differences between female (*p*<0.001) and male (*p*<0.001). The baseline characteristics of the included patients are shown in Table 1 and Supplementary Table 1.

**Table 1:** Baseline characteristics of participants by NSE level.

**Index**	**All patients (N=442)**	**Normal NSE group (N=361)**	**High NSE group (N=81)**	***P* value**
**Basic information**				
Gender (female) (%)	205 (46.38)	171(47.37)	34(41.98)	0.379
Age (years)	73.11±10.80	72.39±10.51	76.33±11.53	0.003
Disease duration (hours)	7.00 (4.00-17.75)	7.00 (3.50-18.00)	7.00 (5.00-15.50)	0.481
**Medical history**				
Hypertension (%)	392 (88.69)	317 (87.81)	75 (92.59)	0.220
Diabetes (%)	156 (35.29)	130 (36.01)	26 (32.10)	0.505
CHD (%)	124 (28.05)	94 (26.04)	30 (37.04)	0.046
Atrial fibrillation (%)	14 (3.17)	9 (2.49)	5 (6.17)	0.174
**Blood routine indicators**				
RBC (10^12^/L)	4.39±0.63	4.39±0.59	4.36±0.77	0.684
RDW-SD (fL)	42.01±2.83	41.67±2.45	43.49±3.80	<0.001
RDW-SD male (fL)	41.93±2.38	41.67±2.15	42.99±2.94	<0.001
RDW-SD female (fL)	42.09±3.27	41.68±2.75	44.18±4.70	<0.001
Average volume of RBC (fL)	90.20±5.15	90.33±5.05	89.59±5.57	0.239
WBC (10^12^/L)	7.09±2.36	6.97±2.28	7.66±2.62	0.017
Neutral cells (10^9^/L)	4.63±2.00	4.54±1.98	5.05±2.02	0.040
Platelets (10^9^/L)	219.96±69.02	217.21±64.90	232.19±84.36	0.137
**Blood biochemical indicators**				
Total cholesterol (mmol/L)	4.45±1.09	4.41±1.09	4.66±1.10	0.062
Triglycerides (mmol/L)	1.35±0.89	1.34±0.93	1.36±0.70	0.920
LDL (mmol/L)	2.82±0.99	2.78±0.99	3.02±1.00	0.043
Fasting blood sugar (mmol/L)	6.16±2.20	6.12±2.10	6.37±2.63	0.351
Alanine aminotransferase (g/L)	17.05 (12.20-24.20)	17.40 (3.30-146.40)	16.30 (3.50- 63.70)	0.211
Creatine (μmol/L)	72.10 (58.62-89.60)	71.40 (58.00-89.00)	77.50 (64.90-93.10)	0.022
Uric acid (μmol/L)	290.87±109.00	288.91±102.01	299.60±136.26	0.507
Homocysteine (μmol/L)	22.00 (16.00-28.00)	21.00 (15.00-28.00)	24.00 (17.00-34.00)	0.071
**Clotting index**				
Prothrombin time (second)	11.00±0.88	10.93±0.77	11.31±1.23	0.008
D-dipolymer (μg/mL)	0.41 (0.23-0.87)	0.38 (0.23-0.81)	0.43 (0.27-1.17)	0.080
**CNS damage index**				
NSE (ng/mL)	13.56±3.26	12.41±2.19	18.69±2.10	<0.001
NSE male (ng/mL)	13.63±3.36	12.39±2.22	18.68±2.23	<0.001
NSE female (ng/mL)	13.47±3.15	12.43±2.15	18.71±1.92	<0.001
**Tumor indicators**				
CEA (ng/mL)	1.48 (0.89-2.34)	1.53 (0.92-2.34)	1.32 (0.87-2.33)	0.251
AFP (ng/mL)	1.80 (1.00-2.80)	1.90 (1.00-2.90)	1.60 (0.70-2.60)	0.152
NSCLC (ng/mL)	2.87 (2.21-3.78)	2.87 (2.21-3.74)	2.83 (2.24-3.89)	0.688
**Medication use before admission**				
Antihypertensive drugs (%)	361 (81.67)	290 (80.33)	71 (87.65)	0.124
Antidiabetic drugs (%)	146 (33.03)	122 (33.80)	24 (29.63)	0.471
Lipid-lowering drugs (%)	212 (47.96)	166 (45.98)	46 (56.79)	0.079
Anticoagulant drugs (%)	7 (1.58)	5 (1.39)	2 (2.47)	0.617
Antiplatelet drugs (%)	388 (87.78)	318 (88.09)	70 (86.42)	0.679

NSE: Neuron-specific enolase, CHD: Coronary heart disease, RBC: Red blood cell, RDW-SD: Red blood cell distribution width -standard deviation, WBC: White blood cell, LDL: Low density lipoprotein, CNS: Central nervous system, CEA: carcinoembryonic antigen, AFP: alpha-fetoprotein, NSCLC: Non-small cell lung cancer-related antigens.

### Comparison of serum NSE levels according to the RDW-SD

**Figure 2:**
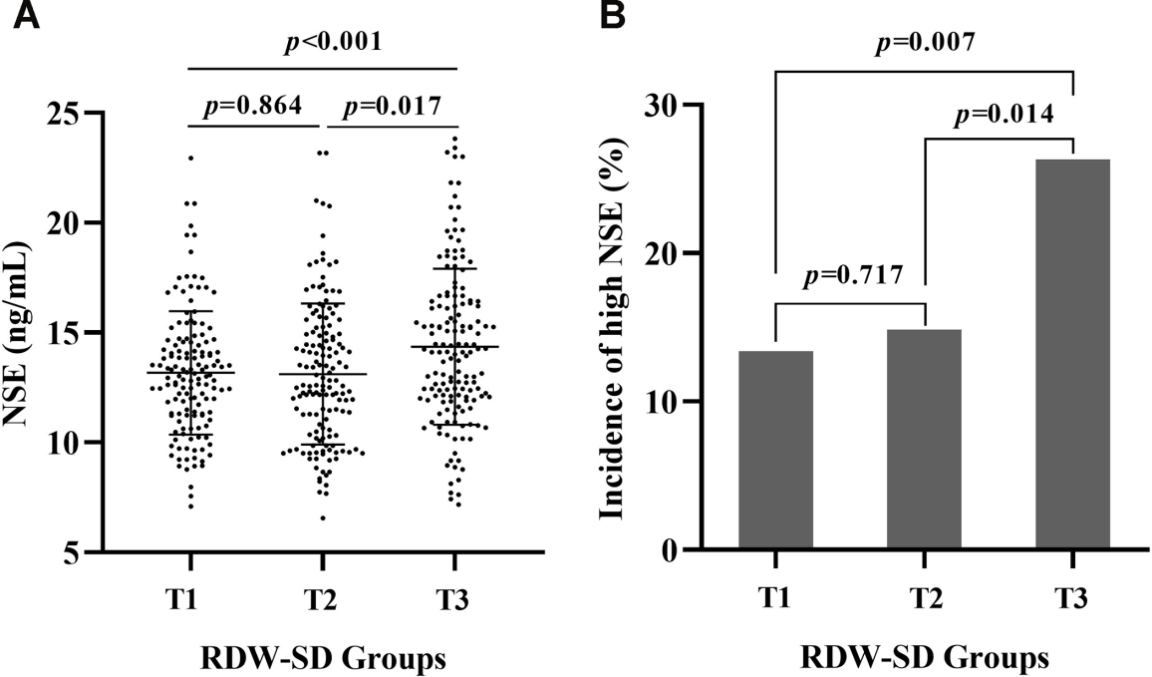
**The serum NSE level and the incidence of high serum NSE according to RDW-SD.** The mean serum NSE levels were 13.17±2.81 ng/mL, 13.11±3.21 ng/mL, and 14.35±3.55 ng/mL in the first, second, and third RDW-SD tertiles in all patients, respectively, and there was a significant difference among the three groups (*p*<0.001) (**A**). The prevalence of high NSE was 19/142 (13.38%), 22/148 (14.86%), and 40/152 (26.32%) in the first, second, and third RDW-SD tertiles, respectively, in all patients, and the prevalence of high NSE showed a grade increase according to the levels of the RDW-SD tertiles (*p*=0.007) (**B**).

The mean RDW-SD levels were 39.41±1.10 fL, 41.51±0.52 fL, and 44.91±2.63 fL in the first, second, and third RDW-SD tertiles for all patients (*p*<0.001). The mean serum NSE levels were 13.17±2.81 ng/mL, 13.11±3.21 ng/mL, and 14.35±3.55 ng/mL in the first, second, and third RDW-SD tertiles for all patients, respectively, and there was a significant difference among the three groups (*p*<0.001). The incidences of high NSE were 19/142 (13.38%), 22/148 (14.86%), and 40/152 (26.32%) in the first, second, and third RDW-SD tertiles in all patients, respectively, and the incidence of high NSE showed a grade increase according to the levels of the RDW-SD tertiles (*p*=0.007). The serum NSE level and the prevalence of high NSE in the first, second, and third RDW-SD tertiles in all patients are shown in Figure 2.

### Relationship between RDW-SD and serum NSE level

**Figure 3:**
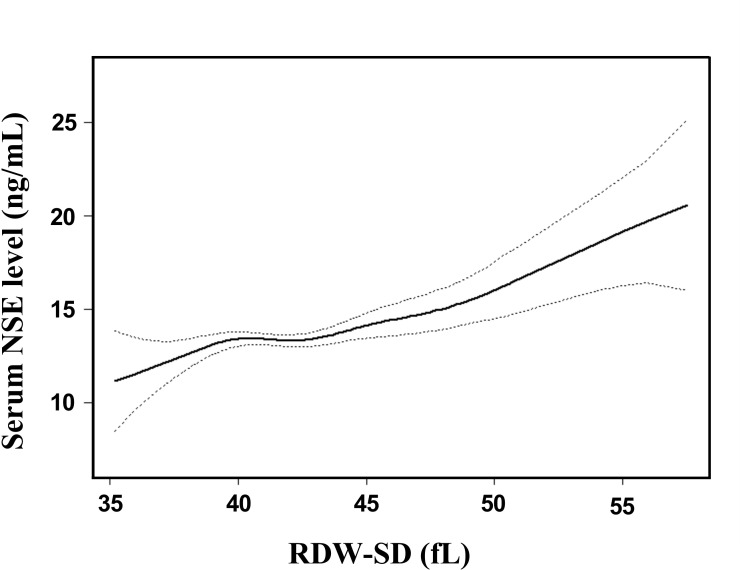
**Association between RDW-SD and serum NSE level.** A linear relationship between RDW-SD and serum NSE level was detected after adjusting for gender, age, hypertension, diabetes, CHD, neutral cells, platelets, LDL, creatine, homocysteine, prothrombin time, antihypertensive drugs, lipid-lowering drugs, and antiplatelet drugs. Solid lines represent the fitting curve, and dotted lines represent the corresponding 95% CI.

Adjusted smoothed plots suggest that there are linear relationships between the RDW-SD and the serum NSE level (Figure 3). The Pearson’s correlation coefficients (95%) for the relationship between RDW-SD and serum NSE level were 0.236 (0.112-0.353, *p*<0.001) in male, 0.321 (0.193-0.439, *p*<0.001) in female, and 0.275 (0.187-0.359, *p*<0.001) in all included patients. The beta coefficient (95%) of RDW-SD was 0.32 (0.21-0.42, *p*<0.001) for all included patients. When multiple linear regression analysis was performed after adjusting for gender, age, hypertension, diabetes, coronary heart disease (CHD), neutral cells, platelets, creatine, homocysteine, prothrombin time, antihypertensive drugs, lipid-lowering drugs, and antiplatelet drugs, the beta coefficient (95%) of RDW-SD was 0.26 (0.15-0.38, *p*<0.001) in all included patients, which showed that the association between RDW-SD and serum NSE level was statistically significant. When RDW-SD was categorized into two groups by RDW-SD 80% quartile levels, the beta coefficients (95%) of high RDW-SD were 2.01 (1.27-2.76, *p*<0.001) and 1.56 (0.78, 2.34, *p*<0.001) in all included patients, which showed that the statistical significance was maintained (Table 2). Hierarchical analysis, according to gender, age, hypertension, diabetes, CHD, neutral cells, platelets, creatine, homocysteine, prothrombin time, antihypertensive drugs, lipid-lowering drugs, and antiplatelet drugs, also showed that the association between RDW-SD and serum NSE level was statistically significant (Supplementary Table 2).

**Table 2:** Association between RDW-SD and NSE.

**Variable**	**Model 1 (unadjusted)**		**Model 2 (adjusted)**	
	**N**	**β (95% CI) *p***	**N**	**β (95% CI) *p***
RDW-SD Total (continuous)	442	0.32 (0.21, 0.42) <0.001	442	0.26 (0.15, 0.38) <0.001
RDW-SD Total (categorical)	442		442	
RDW-SD (<80%)	355	ref	355	ref
RDW-SD (≥80%)	87	2.01 (1.27, 2.76) <0.001	87	1.56 (0.78, 2.34) <0.001
RDW-SD Tertile (categorical)	442		442	
Tertile 1	142	ref	142	ref
Tertile 2	148	-0.06 (-0.80, 0.68) 0.872	148	-0.28 (-1.03, 0.46) 0.457
Tertile 3	152	1.18 (0.45, 1.92) 0.002	152	0.93 (0.33, 1.53) 0.015

Model 1: unadjusted.

Model 2: adjusted for gender, age, hypertension, diabetes, CHD, neutral cells, platelets, creatine, homocysteine, prothrombin time, antihypertensive drugs, lipid-lowering drugs, and antiplatelet drugs.

In this model, the beta coefficients (95% CI) of other covariates were as follows: gender (female) -0.16 (-0.17, -0.15), *p*=0.016; age (per 1 yr), 0.03 (0.00, 0.06), *p*=0.022; hypertension 0.44 (0.32, 0.56), *p*=0.036; diabetes -0.22 (-0.29, -0.15), *p*=0.049; CHD 0.62 (0.56, 0.68), *p*=0.073; neutral cells 0.18 (0.03, 0.34), *p*=0.017; platelets 0.01 (0.00, 0.01), *p*=0.011; creatine 0.01 (0.00, 0.02), *p*=0.008; homocysteine 0.03 (0.01, 0.06), *p*=0.005; prothrombin time 0.48 (0.14, 0.82), *p*=0.005; antihypertensive drugs 0.17 (0.11, 0.23), *p*=0.054; lipid-lowering drugs, 0.21 (0.18, 0.24), *p*=0.118; and antiplatelet drugs, -0.32 (-0.43, -0.21), *p*=0.503.

## DISCUSSION

Our study found a positive correlation between the RDW-SD and serum NSE level, and the NSE level and incidence of high NSE were increased with graded RDW-SD in both genders. Furthermore, the association between RDW-SD and serum NSE level was independent after adjustment for other confounding risk factors and hierarchical analysis. Therefore, these results demonstrated that RDW-SD was associated with neuronal damage in AIS patients.

Currently, NSE is used as a biomarker to assess neuronal damage and to predict stroke prognosis. The determination of NSE in serum and CSF after cerebral ischemia and brain injury provides a reliable laboratory index for determining the degree of neuronal injury [18, 19]. During the process of ischemic stroke, the blood-brain barrier is disrupted by endothelial cell death with neuronal degeneration, neurogliocyte swelling and necrosis. Subsequently, the NSE released from injured neurons passes through the blood-brain barrier and spreads rapidly through the ischemic tissue to the bloodstream [20]. Then, NSE accumulates at the early stage of the ischemic cascade in blood, where it has a biological half-life of approximately 48 hours [21, 22]. Previous studies have shown that serum NSE increases beginning at 2 hours, peaks after the first 6-24 hours, and persists for 1-7 days [23–25]. The first NSE peak occurs within 7-18 hours after stroke, and it may be attributed to the initial damage of neuronal tissue, while the second NSE peak, between days 2–4, may reflect the secondary neuronal damage caused by edema and an increase in intracranial pressure [26]. Other studies showed an increase in serum NSE levels after acute ischemia in humans and no significant differences in serum NSE levels between days 1, 2, and 3 [27]. Therefore, previous studies provide strong evidence for our study to apply NSE levels to evaluate neuronal damage. In our study, included ischemic stroke patients were acute onset within 48 hours, and serum NSE levels were measured upon admission. This ensures the accuracy of the NSE levels so as to reliably assess the neuronal damage.

Our study revealed that RDW was associated with serum NSE levels in AIS patients. The mechanism has not yet been fully clarified, but it may be related to many factors, the first of which is hemodynamic changes. With the increase in RDW, the size of red blood cells (RBCs) is heterogeneous, and their deformation causes functional changes in peripheral blood circulation, which may be an independent or synergistic factor increasing in circulatory resistance and leading to vascular occlusion [28]. In addition, the increased RDW can also promote platelet activation and aggregation [29]. Second, the inflammatory reaction increased. Studies have shown that increased IL-18 level contributed to the development of ischemic stroke, and nonspecific inflammatory factors, such as hypersensitivity C-reactive protein and ESR, are elevated in the high RDW group [30, 31]. Similarly, in our study, we observed elevated RDW-SD levels in high NSE level patients, with an increase in ESR, white blood cell (WBC) count and number of neutral cells. Excessive inflammatory reactions produced large amounts of reactive oxygen and pro-inflammatory cytokines, which increased the clonal hematopoiesis of indeterminate potential (CHIP) in peripheral-blood cells [32]. CHIP was associated with nearly a doubling in the risk of coronary heart disease in humans and with accelerated atherosclerosis in mice [33]. Therefore, the inflammatory reaction is closely related to RDW-SD levels and neuronal damage. Another factor may be lipid metabolism disorders. A previous study found that patients with high RDW tend to have complications related to lipid metabolism, and lipid metabolism disorders have been confirmed to have a close correlation with the severity of the disease in AIS patients [34]. In our study, we also observed elevated levels of blood lipids, especially low density lipoprotein (LDL). Finally, oxidative stress response may be a factor. Increased RDW is also related to oxidative stress and the release of cytokines in response to inflammation [35]. Oxidative stress induced the adhesion of erythrocytes to the vascular endothelium and reduced the deformability of erythrocytes [36]. Therefore, oxidative stress may be a critical factor involved in high RDW leading to ischemic stroke.

Several limitations should be considered in the interpretation of our results. First, the disease duration before admission in our study ranged from 0.5-46 hours, with a median and interquartile range of 7 (4-17.75) hours, and the time to venous blood sample collection after onset may confound our findings of serum NSE levels; therefore, further research should include information on patients between 12 and 36 hours after early onset. Second, the enrolled subjects of our study were AIS patients, and some patients had accompanying potential risk factors of cerebral infarction and took antiplatelet, antihypertensive and hypoglycemic drugs before admission. Although we included medication use before admission in the analysis, we could still not completely exclude the influence of concomitant medication on RDW-SD. The use of drugs may confound our findings; thus, further research needs to examine the association between RDW-SD and serum NSE levels in AIS patients without other diseases. Despite these noted limitations, there are several strong points of this study. Our analyzed data were obtained from a large sample of the AIS population, which can guarantee reliable conclusions. Additionally, all studied patients excluded malignancies, heart failure, anemia, erythrocytosis, hepatic and renal diseases, and currently using supplements including iron, folic acid, and vitamin B12 that may cause abnormal values of NSE and RBC; further, the assessed tumor indicators for patient were within the normal range. This ensures the accuracy of the data, and we rigorously managed the data collection to arrive at a more reliable conclusion.

In conclusion, this study demonstrates that RDW-SD was associated with serum NSE levels in AIS patients, and RDW-SD could be considered a risk factor for neuronal damage in the AIS population. Further studies are required that have a larger number of patients and that further define the value of RDW-SD in neuronal damage in the AIS population.

## MATERIALS AND METHODS

### Ethics

This study was performed according to the principles of the Declaration of Helsinki and was approved by the ethics committee of Baoshan Branch, Renji Hospital, School of Medicine, Shanghai Jiaotong University, Shanghai, China. We obtained informed consent from all patients or their immediate family members prior to sample collection.

### Design

This was a cross-sectional study designed to explore the correlation between RDW-SD and serum NSE levels in the AIS population. Consecutive patients with AIS were enrolled in our study from the Baoshan Branch of Renji Hospital in China from January 2018 to June 2019, and patient data were recorded in the Stroke Registry Database of the hospital.

### Study subjects

Patients were diagnosed with AIS according to the criteria defined by the World Health Organization criteria [37]. The inclusion criteria were (1) acute onset of ischemic stroke within 48 hours, (2) focal signs of cerebral dysfunction persisting after acute onset, (3) confirmation by computed tomography (CT) or magnetic resonance imaging (MRI) of the brain within 24 hours after admission; follow-up CT or MRI was performed within 14 days of admission or in any case of neurological deterioration, and (4) aged ≥40 yr.

The exclusion criteria were (1) intracerebral hemorrhage, (2) transient ischemic attack, (3) malignancies, (4) heart failure, anemia, erythrocytosis, hepatic and renal disease, (5) currently using supplements, including iron, folic acid, and vitamin B12, and (6) data were not available for review, including unintegrated clinical and laboratory data.

### Clinical and laboratory data

The demographic data, medical history [hypertension, diabetes, CHD and atrial fibrillation], and medication used before admission (antihypertensive drugs, antidiabetic drugs, lipid-lowering drugs, anticoagulant drugs, and antiplatelet drugs) were collected upon admission via in-person interviews with the patients or their family members.

Fasting venous blood samples were collected upon admission and prior to drug administration, such as intravenous tissue plasminogen activator, or any intraarterial revascularization procedure in the emergency room. Serum NSE levels were measured with commercially available quantitative enzyme-linked immunosorbent assay kits obtained from R&D Systems (Shanghai, China). Intra- and interassay coefficients of variation were b=3% and b=7%, respectively. The minimum detection limit was 0.229 ng/mL, and the detection values ranged from 0.625 to 40 ng/ml for NSE. The NSE reference value in our laboratory was < 16.3 mg/L. Blood samples were also collected to measure routine blood indicators [RBC count, RDW-SD, average volume of RBC, WBC count, neutral cell count, and platelet count], blood biochemical indicators [levels of total cholesterol, triglycerides, LDL, fasting blood sugar, alanine aminotransferase, creatine, uric acid, and homocysteine], clotting index (prothrombin time and D-dipolymer), and tumor indicators [levels of carcinoembryonic antigen (CEA) and alpha-fetoprotein (AFP), and presence of non-small cell lung cancer (NSCLC)]. All of the above determinations were performed in the hospital’s laboratory by individuals blinded to the clinical data.

### Groups

The patients in our study were grouped according to two criteria. 1), First, groups were organized by serum NSE level. The normal range of serum NSE levels was less than 16.3 ng/mL; thus, high NSE was identified when its value was greater than or equal to 16.3 ng/mL, and included patients were categorized into a group with normal NSE (0 to 16.3 ng/mL) and a group with high NSE levels (greater than or equal to 16.3 ng/mL). 2), Second, groups were organized by RDW-SD. Patients were categorized into two groups by RDW-SD 80% quartile levels (RDW-SD<80% group and RDW-SD≥80% group). Patients were also categorized into the T1, T2 and T3 groups according to the RDW-SD tertile levels. In addition, patients were also grouped based on the clinical normal reference value of indicators in hierarchical analysis.

### Statistical analysis

Categorical variables were presented as counts and percentages and were analyzed by Fisher’s exact tests or chi-square tests. Continuous variables were reported as the means and standard deviations for data of normal distribution, which were analyzed by *t* tests, and they were reported as medians and interquartile ranges for data of abnormal distribution, which were analyzed by Mann-Whitney U tests. The association between RDW-SD and serum NSE level was assessed by Pearson’s correlation analysis and multiple linear regression analysis. Both non-adjusted and multivariate adjusted models were applied, and interaction and stratified analyses were conducted. Statistical analyses were performed using Statistical Package of the Social Sciences Software version 21.0 (SPSS, Chicago, IL, USA), and statistical graphics were generated using GraphPad PRISM 5 (Graph Pad Software Inc., San Diego, CA, USA). The level of significance was set with a two-tailed *p*-value of <0.05.

## Supplementary Material

Supplementary Tables
