# Supplementary material for: Red blood cell distribution width is associated with neuronal damage in acute ischemic stroke
Source: Aging (Albany NY). 2020 May 23;12(10):9855–67. doi: 10.18632/aging.103250 (PMC7288978; doi:10.18632/aging.103250)
Supplement: Supplementary Tables [file aging-12-103250-s001..pdf]

SUPPLEMENTARY TABLES

Supplementary Table 1. Baseline characteristics of participants by RDW-SD level.

Variables	RDW-SD (fL)			*P*-value
	Tertile 1 (N=142)	Tertile 2 (N=148)	Tertile 3 (N=152)	
**Basic information**				
Gender (female) (%)	76 (53.52)	58 (39.19)	71 (46.71)	0.050
Age (years)	69.41±9.72	72.45±10.46	77.22±10.75	<0.001
Disease duration (hours)	6.50 (3.00-15.75)	7.00 (4.38-18.25)	8.00 (3.88-17.00)	0.319
**Medical history**				
Hypertension (%)	124 (87.32)	135 (91.22)	133 (87.50)	0.492
Diabetes (%)	64 (45.07)	49 (33.11)	43 (28.29)	0.009
CHD (%)	29 (20.42)	40 (27.03)	55 (36.18)	0.010
Atrial fibrillation (%)	1 (0.70)	4 (2.70)	9 (5.92)	0.030
**Blood routine indicators**				
RBC (10^{12}/L)	4.50±0.57	4.46±0.63	4.20±0.65	<0.001
RDW-SD (fL)	39.41±1.10	41.51±0.52	44.91±2.63	<0.001
RDW-SD male (fL)	39.50±1.17	41.46±0.47	44.44±1.96	<0.001
RDW-SD female (fL)	39.34±1.03	41.59±0.58	45.45±3.15	<0.001
Average volume of RBC (fL)	88.40±5.52	91.06±4.31	91.03±5.16	<0.001
WBC (10^{12}/L)	6.86±1.98	7.15±2.26	7.26±2.74	0.320
Neutral cells (10^{9}/L)	4.37±1.76	4.68±2.06	4.84±2.13	0.127
Platelets (10^{9}/L)	224.06±58.03	219.18±68.51	216.88±78.56	0.664
**Blood biochemical indicators**				
Total cholesterol (mmol/L)	4.47±1.10	4.39±1.10	4.49±1.09	0.695
Triglycerides (mmol/L)	1.16 (0.81-1.61)	1.13 (0.87-1.69)	1.17 (0.84-1.49)	0.902
LDL (mmol/L)	2.79±0.96	2.78±1.00	2.89±1.01	0.590
Fasting blood sugar (mmol/L)	6.59±2.65	6.17±2.25	5.76±1.53	0.005
Alanine aminotransferase (g/L)	17.50 (12.90-24.20)	18.35 (13.28-24.42)	16.10 (10.93-22.22)	0.069
Creatine (μmol/L)	73.40 (57.10-89.72)	72.60 (60.32-88.48)	71.40 (58.42-89.60)	0.995
Uric acid (μmol/L)	287.14±95.20	305.70±96.29	279.91±129.84	0.108
Homocysteine (μmol/L)	15.00 (9.00-22.00)	17.50 (11.00-23.00)	18.00 (12.00-25.00)	0.037
**Clotting index**				
Prothrombin time (second)	10.82±0.73	10.95±0.85	11.21±1.00	<0.001
D-dipolymer (μg/mL)	0.36 (0.20-0.63)	0.36 (0.22-0.69)	0.54 (0.30-1.40)	<0.001
**CNS damage index**				
Incidence of high NSE (%)	19 (13.38)	22 (14.86)	40 (26.32)	0.007
NSE (ng/mL)	13.17±2.81	13.11±3.21	14.35±3.55	<0.001
NSE male (ng/mL)	13.36±3.03	13.22±3.16	14.32±3.73	0.075
NSE female (ng/mL)	13.01±2.61	12.95±3.31	14.40±3.37	0.009
**Tumor indicators**				
CEA (ng/mL)	1.33 (0.75-2.18)	1.55 (0.99-2.42)	1.55 (1.00-2.33)	0.031
AFP (ng/mL)	1.80 (1.00-2.60)	2.00 (0.97-3.40)	1.70 (0.80-2.73)	0.188
NSCLC (ng/mL)	2.88 (2.27-3.74)	2.98 (2.21-3.82)	2.79 (2.16-3.74)	0.579
**Medication use before admission**				
Antihypertensive drugs (%)	112 (78.87)	126 (85.14)	123 (80.92)	0.370
Antidiabetic drugs (%)	59 (41.55)	47 (31.76)	40 (26.32)	0.020
Lipid-lowering drugs (%)	64 (45.07)	71 (47.97)	77 (50.66)	0.632

Anticoagulant drugs (%)	0 (0.00)	2 (1.35)	5 (3.29)	0.076
Antiplatelet drugs (%)	133 (93.66)	132 (89.19)	123 (80.92)	0.003

NSE: Neuron-specific enolase, CHD: Coronary heart disease, RBC: Red blood cell, RDW-SD: Red blood cell distribution width - standard deviation, WBC: White blood cell, LDL: Low density lipoprotein, CNS: Central nervous system, CEA: carcinoembryonic antigen, AFP: alpha-fetoprotein, NSCLC: Non-small cell lung cancer-related antigens.

Supplementary Table 2. Hierarchical analysis of the relationship of RDW-SD and NSE.

Hierarchical factor	N	Model 1 (unadjusted)	Model 2 (adjusted)	*p* for interaction
		β (95% CI) *p*	β (95% CI) *p*	
Gender				
Female	205	0.31 (0.18, 0.43) <0.001	0.27 (0.09, 0.45) 0.003	0.915
Male	237	0.33 (0.16, 0.51) <0.001	0.27 (0.09, 0.44) 0.002	
Age (years)				
<75	246	0.20 (0.02, 0.38) 0.032	0.19 (0.01, 0.37) 0.040	0.285
≥75	196	0.35 (0.22, 0.49) <0.001	0.31 (0.17, 0.45) <0.001	
Hypertension				
No	50	0.40 (0.04, 0.77) 0.030	0.40 (0.03, 0.77) 0.035	0.441
Yes	392	0.31 (0.20, 0.42) <0.001	0.25 (0.14, 0.37) <0.001	
Diabetes				
No	286	0.34 (0.21, 0.46) <0.001	0.30 (0.17, 0.44) <0.001	0.313
Yes	156	0.28 (0.11, 0.46) 0.001	0.19 (0.01, 0.37) 0.042	
CHD				
No	318	0.28 (0.15, 0.42) <0.001	0.24 (0.10, 0.38) <0.001	0.583
Yes	124	0.35 (0.18, 0.52) <0.001	0.30 (0.12, 0.48) 0.001	
Neutral cells (10^9/L)				
<6.3	377	0.28 (0.16, 0.40) <0.001	0.23 (0.11, 0.36) <0.001	0.259
≥6.3	65	0.42 (0.20, 0.64) <0.001	0.38 (0.15, 0.60) 0.001	
Platelets (10^9/L)				
<300	386	0.33 (0.22, 0.44) <0.001	0.25 (0.13, 0.37) <0.001	0.596
≥300	56	0.19 (-0.11, 0.48) 0.219	0.17 (-0.13, 0.47) 0.265	
Creatine (μmol/L)				
<106	384	0.29 (0.17, 0.41) <0.001	0.22 (0.09, 0.35) <0.001	0.207
≥106	58	0.41 (0.20, 0.61) <0.001	0.38 (0.16, 0.59) <0.001	
Homocysteine (μmol/L)				
<15	174	0.21 (0.01, 0.41) 0.036	0.21 (0.01, 0.41) 0.042	0.492
≥15	268	0.35 (0.22, 0.47) <0.001	0.29 (0.16, 0.43) <0.001	
Prothrombin time (second)				
<11	240	0.12 (-0.03, 0.27) 0.125	0.07 (-0.10, 0.23) 0.430	<0.001
≥11	202	0.47 (0.33, 0.60) <0.001	0.43 (0.29, 0.57) <0.001	
Antihypertensive drugs				
No	81	0.31 (0.05, 0.58) 0.021	0.26 (-0.01, 0.53) 0.062	0.975
Yes	361	0.31 (0.20, 0.42) <0.001	0.26 (0.14, 0.38) <0.001	
Lipid-lowering drugs				
No	230	0.35 (0.21, 0.49) <0.001	0.29 (0.14, 0.43) 0.001	0.611
Yes	212	0.27 (0.12, 0.43) <0.001	0.23 (0.07, 0.39) 0.004	
Antiplatelet drugs				

No	54	0.24 (-0.09, 0.57) 0.153	0.18 (-0.15, 0.51) 0.288	0.592
Yes	388	0.33 (0.22, 0.44) <0.001	0.27 (0.15, 0.39) <0.001	

Model 1: unadjusted.

Model 2: Each stratification adjusted for all the factors (gender, age, hypertension, diabetes, CHD, neutral cells, platelets, creatine, homocysteine, prothrombin time, antihypertensive drugs, lipid-lowering drugs, and antiplatelet drugs) except for the stratification factor itself.
